# Genomic insights into lineage-specific evolution of the *oleosin* family in Euphorbiaceae

**DOI:** 10.1186/s12864-022-08412-z

**Published:** 2022-03-05

**Authors:** Zhi Zou, Yongguo Zhao, Li Zhang

**Affiliations:** 1 Hainan Key Laboratory for Biosafety Monitoring and Molecular Breeding in Off-Season Reproduction Regions, Institute of Tropical Biosciences and Biotechnology/Sanya Research Institute of Chinese Academy of Tropical Agricultural Sciences, Haikou, 571101 Hainan People’s Republic of China; 2 Guangdong University of Petrochemical Technology, Maoming, 525000 Guangdong People’s Republic of China; 3 Hubei Provincial Key Laboratory for Protection and Application of Special Plants in Wuling Area of China, College of Life Science, South-Central University for Nationalities, Wuhan, 430074 Hubei People’s Republic of China

**Keywords:** Collinear relationship, Evolution, Expansion, Expression divergence, Ortholog group, Phylogenetic analysis, Whole-genome duplication

## Abstract

**Background:**

Lipid droplets (LDs) present in land plants serve as an essential energy and carbon reserve for seed germination and seedling development. Oleosins, the most abundant structural proteins of LDs, comprise a small family involved in LD formation, stabilization and degradation. Despite their importance, our knowledge on oleosins is still poor in Euphorbiaceae, a large plant family that contains several important oil-bearing species.

**Results:**

To uncover lineage-specific evolution of *oleosin* genes in Euphorbiaceae, in this study, we performed a genome-wide identification and comprehensive comparison of the *oleosin* family in Euphorbiaceae species with available genome sequences, i.e. castor bean (*Ricinus communis*), physic nut (*Jatropha curcas*), tung tree (*Vernicia fordii*), *Mercurialis annua*, cassava (*Manihot esculenta*) and rubber tree (*Hevea brasiliensis*), and a number of five, five, five, five, eight and eight members were found, respectively. Synteny analysis revealed one-to-one collinear relationship of *oleosin* genes between the former four (i.e. castor bean, physic nut, tung tree and *M. annua*) as well as latter two species (i.e. cassava and rubber tree), whereas one-to-one and one-to-two collinear relationships were observed between physic nut and cassava, reflecting the occurrence of one recent whole-genome duplication (WGD) in the last common ancestor of cassava and rubber tree. The presence of five ortholog groups representing three previously defined clades (i.e. U, SL and SH) dates back at least to the Malpighiales ancestor, because they are also conserved in poplar (*Populus trichocarpa*), a tree having experienced one Salicaceae-specific recent WGD. As observed in poplar, WGD was shown to be the main driver for the family expansion in both cassava and rubber tree. Nevertheless, same retention patterns of WGD-derived duplicates observed in cassava and rubber tree are somewhat different from that of poplar, though certain homologous fragments are still present in rubber tree. Further transcriptional profiling revealed an apparent seed-predominant expression pattern of *oleosin* genes in physic nut, castor bean and rubber tree. Moreover, structure and expression divergence of paralogous pairs were also observed in both cassava and rubber tree.

**Conclusion:**

Comparative genomics analysis of *oleosin* genes reported in this study improved our knowledge on lineage-specific family evolution in Euphorbiaceae, which also provides valuable information for further functional analysis and utilization of key members and their promoters.

**Supplementary Information:**

The online version contains supplementary material available at 10.1186/s12864-022-08412-z.

## Background

Euphorbiaceae (spurge), which belongs to the order Malpighiales, is a very large family composed of more than 7000 species in around 300 genera. They appear as herbs, shrubs, and trees that are widely distributed in tropical, subtropical, and temperate regions [1]. The economic importance has prompted active attempts on genome characterization of several Euphorbiaceae species, i.e., castor bean (*Ricinus communis*), physic nut (*Jatropha curcas*), rubber tree (*Hevea brasiliensis*), cassava (*Manihot esculenta*), tung tree (*Vernicia fordii*), and *Mercurialis annua* [2–10]. Among them, *M. annua*, a wind-pollinated annual herb originated in Europe, North Africa, and Middle East, represents an ideal model plant for studying sexual systems [10]. Castor bean, physic nut, and tung tree, which are native to Africa, Central America, and China, respectively, are three important non-food oilseed shrubs or small trees accumulating a high level of oil (>40%) in their seeds. The physic nut oil with fossil fuel-like fatty acid composition is a potential material for biodiesel production; the castor oil dominant in ricinoleic acid is widely used for industrial, medicinal, and cosmetic purposes; and, the tung oil rich in α-eleostearic acid (α-ESA) is widely used in the production of inks, dyes, resins, and biodiesel [2, 5, 9]. Cassava and rubber tree, both of which originated in the Southern Amazon basin, also accumulate more than 25% of oil in their seeds, though they have not been well explored [11]. Instead, the starchy-enriched storage roots of cassava are not only staple food for millions of people but also ideal for bio-ethanol production, whereas natural rubber or *cis*-1,4-polyisoprene, which is specifically produced by the rubber tree laticifer, is an indispensable industrial raw material for various uses [6, 12]. Despite the diversity in morphology and traits of cassava and rubber tree, they were proven to share one so-called ρ whole-genome duplication (WGD) event after the split with other Euphorbiaceae plants, occurred within a window of 39–47 million years ago (Mya) [6, 13–15]. In evolutionary terms, it is of particular interest to study species-specific evolution of genes associated with certain economic traits in Euphorbiaceae.

In plants, lipids in the form of triacylglycerols (TAGs) are the most abundant energy-dense storage compounds in seeds as well as several vegetative tissues [16]. TAGs are stored within lipid droplets (LDs) or oil bodies (OBs) that are characterized by a layer of phospholipids and several types of structural proteins such as oleosins, caleosins, and steroleosins [17]. Oleosins, the small (14–30 kDa) but most abundant LD proteins, feature a conserved central hydrophobic portion that is known as the proline knot motif (−PX_5_SPX_3_P-) of approximately 72 residues, whereas N- and C-terminal peptides are amphipathic and usually variable [18, 19]. *Oleosin* genes are widely distributed from single-celled algae to land plants. In contrast to a single or few members found in green algae, the *oleosin* family is highly abundant and diverse in land plants [19–21]. For example, there are six, six, 13, 17 or 48 members present in safflower (*Carthamus tinctorius*), rice (*Oryza sativa*), flax (*Linum usitatissimum*), arabidopsis (*Arabidopsis thaliana*), and rapeseed (*Brassica napus*), respectively [20, 22–25]. Based on sequence similarity, oleosins could be divided into five clades: the P clade, which represents the primitive one, is only found in green algae, mosses, and ferns; the U clade is universally present in all land plants; and, another three clades, i.e., SL, SH, and T, are organ-specific [19]. The SL clade represents low-molecular-weight peptides that are present in seeds of gymnosperms and angiosperms; the SH clade are high-molecular-weight peptides present in seeds of angiosperms; and, the T clade is tapetum-specific of the Brassicaceae lineage [19, 26]. Whereas LDs serve as an essential energy and carbon reserve for seed germination and seedling development, oleosins function in LD formation, stabilization, and degradation [27, 28]. An exciting fact is that oleosins are directly involved in regulating LD size and overexpression of *oleosin* genes could increase the seed oil content in arabidopsis [24, 25, 29, 30]. Moreover, a recent study revealed that strong artificial selection of *GmOLEO1*, which resulted in its high expression and increased seed oil accumulation in cultivated relatives, had occurred during soybean (*Glycine max*) domestication [31]. In castor bean, four *oleosin* genes have previously been described [23], and two of them, which represent major seed LD proteins of 14 and 16 kDa, respectively, have also been characterized *via* MALDI-MS and CID tandem MS [32]. In tung tree, mining transcriptome data resulted in five *oleosin* genes, which were shown to preferentially express in developing seeds relative to leaves and flowers [33]. Nevertheless, *oleosin* genes in other Euphorbiaceae plants and lineage-specific evolution of this special family have not been investigated. To address this issue, in the present study, we took advantage of available genome sequences and transcriptome datasets to identify the complete set of *oleosin* family genes in these Euphorbiaceae plants. Ortholog groups (OGs) and gene expansion patterns were inferred from phylogenetic, best-reciprocal-hit (BRH) BLAST as well as synteny analyses, whereas the evolutionary patterns were investigated based on the analysis of their gene structures, sequence characteristics, conserved motifs, and expression profiles.

## Results

### Identification, chromosome location, and synteny analysis of *oleosin* genes in six Euphorbiaceae plants

**Fig. 1:**
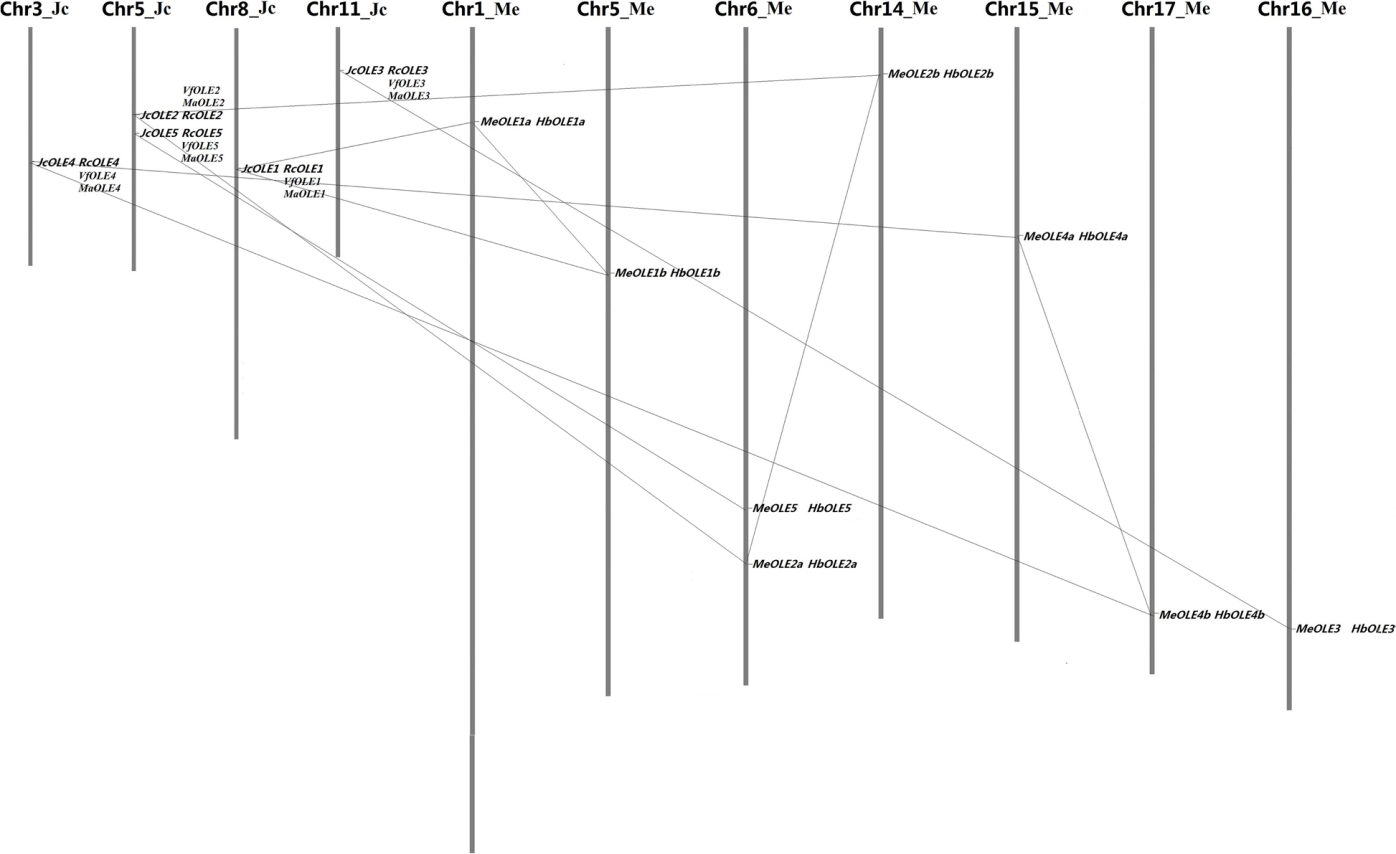
Chromosomal locations and duplication events of *Jc*/*MeOLE* genes and their collinear genes in castor bean/tung tree/*M. annua* and rubber tree, respectively. Chromosome serial numbers are indicated at the top of each chromosome, and lines connect duplicate pairs located within syntenic blocks. Collinear genes in castor bean, *M. annua*, tung tree, and rubber tree are shown just behind that of physic nut and cassava, respectively

According to comparative genomics analyses, physic nut, tung tree, castor bean, and *M. annua* are typical diploid species that didn’t experience recent WGDs after the ancient so-called γ whole-genome triplication shared by core eudicots [2, 6, 9, 10]. In contrast to the fragmented status of genome assemblies in castor bean (25,763 scaffolds) [2], tung tree (20,614 scaffolds) [9], and *M. annua* (74,927 scaffolds) [10], the physic nut genome used in this study is mainly comprised of 6023 scaffolds and 81.7% of this assembly could be anchored onto 11 chromosomes (Chrs) based on genetic markers [6]. As shown in Table 1, a total of five *oleosin* family genes were identified from the physic nut genome, which were named *JcOLE1–5* according to phylogenetic analysis (see below). The expression of these genes was all supported by RNA-seq reads as well as ESTs, which also allowed the extension of their transcription regions. Although *JcOLE* genes are distributed across five scaffolds, they were further anchored onto four pseudochromosomes with the help of the available genetic map, i.e., Chr3, Chr5, Chr8, and Chr11 (Fig. 1).

**Table 1:** *Oleosin* family genes identified in six Euphorbiaceae plants

Gene name	Locus name	Position	EST hits	AA	MW (kDa)	***p***I	AI	GRAVY	Oleosin location	Comment
*JcOLE1*	JCGZ_05847	Scaffold1855:288075..288870(+)	4	163	17.55	10.07	108.40	0.465	37..149	
*JcOLE2*	JCGZ_26302	Scaffold906:2378948..2379800(+)	146	137	14.28	9.65	108.91	0.399	17..129	
*JcOLE3*	JCGZ_07714	Scaffold211:4432780..4433488(−)	3	146	15.73	9.30	97.05	0.334	20..132	
*JcOLE4*	JCGZ_10267	Scaffold27:1667720..1668854(+)	92	155	16.61	9.99	108.84	0.250	32..144	
*JcOLE5*	JCGZ_11426	Scaffold328:794613..795414(+)	37	147	15.58	10.09	103.54	0.354	26..138	
*RcOLE1*	30,174.t000125	Scaffold30174:2548082..2548991(−)	198	169	18.53	9.86	91.83	0.224	43..155	
*RcOLE2*	30,147.t000604	Scaffold30147:1397782..1399509(+)	2	138	14.48	10.11	108.19	0.381	17..129	
*RcOLE3*	29,794.t000071	Scaffold29794:410823..411544(−)	1	148	15.83	9.44	93.65	0.326	20..132	Mis-annotated
*RcOLE4*	29,917.t000061	Scaffold29917:392657..393518(−)	62	153	16.29	9.74	93.14	0.131	31..142	
*RcOLE5*	30,147.t000162	Scaffold30147:3161936..3162665(+)	6	141	14.96	10.96	107.87	0.322	21..133	
*VfOLE1*	–	Scaffold726:116795..117934(+)	0	170	18.31	9.84	107.64	0.231	39..151	
*VfOLE2*	–	Scaffold52:288433..290739(+)	88	134	14.00	9.98	104.60	0.397	17..129	
*VfOLE3*	–	Scaffold468:163522..164278(−)	1	147	15.73	8.75	96.34	0.261	20..132	
*VfOLE4*	–	Scaffold141:355782..356246(−)	18	152	16.18	9.93	104.48	0.152	31..143	
*VfOLE5*	–	Scaffold9:357159..358127(+)	22	148	15.65	10.21	93.76	0.303	32..144	
*MaOLE1*	–	Scaffold76975:68..614(−)	0	170	18.31	9.84	89.00	0.231	44..156	
*MaOLE2*	–	Scaffold5360:5948..6436(+)	0	134	14.00	9.98	94.70	0.397	15..127	
*MaOLE3*	–	Scaffold13777:6055.. 6498(+)	0	147	15.73	8.75	88.44	0.261	19..131	
*MaOLE4*	–	Scaffold75566:1..452(−)	0	152	16.18	9.93	92.43	0.152	29..141	
*MaOLE5*	–	Scaffold64893:268..714(+)	0	148	15.65	10.21	98.85	0.303	23..134	
*MeOLE1a*	Manes.01G021400	Chr01:3581610..3582383(−)	5	200	21.72	10.00	113.85	0.451	74..186	Mis-annotated
*MeOLE1b*	Manes.05G118400	Chr05:11792103..11792947(+)	0	160	17.32	9.74	103.69	0.413	34..146	
*MeOLE2a*	Manes.06G131000	Chr06:23786505..23787260(−)	0	135	14.10	9.90	114.96	0.479	18..130	
*MeOLE2b*	Manes.14G039100	Chr14:3104201..3105039(+)	0	138	14.44	9.61	113.84	0.408	18..130	
*MeOLE3*	Manes.16G076500	Chr16:23230952..23231602(+)	0	150	16.22	9.34	106.73	0.293	21..133	
*MeOLE4a*	Manes.15G149800	Chr15:11772517..11772972(−)	0	151	16.23	7.97	103.38	0.322	26..138	
*MeOLE4b*	Manes.17G112800	Chr17:25320073..25320540(+)	0	155	16.40	9.81	102.65	0.255	32..144	
*MeOLE5*	Manes.06G108600	Chr06:21806141..21806626(−)	0	161	16.82	9.89	94.53	0.136	30..142	
*HbOLE1a*	Scaffold2159_36666	Scaffold2159:36264..37302(+)	1	160	17.34	9.81	106.00	0.417	34..146	
*HbOLE1b*	Scaffold1416_98900	Scaffold1416:98659..99141(−)	0	160	17.45	9.48	109.19	0.475	34..146	
*HbOLE2a*	Scaffold0426_909621	Scaffold0426:909292..910191(+)	0	139	14.42	9.80	113.02	0.386	18..130	
*HbOLE2b*	Scaffold0426_939203	Scaffold0426:939051..939883(−)	0	138	14.23	9.87	119.57	0.515	18..130	
*HbOLE3*	Scaffold0359_76313	Scaffold0359:75823..76778(+)	0	148	15.99	9.43	94.26	0.218	20..132	
–	–	Scaffold0241:224753..224879(+)	–	–	–	–		–	–	Pseudogene
–	–	Scaffold0154:1876201..1876327(−)	–	–	–	–		–	–	Pseudogene
*HbOLE4a*	Scaffold0980_8021	Scaffold0980:7788..8255(−)	0	152	16.06	9.79	107.11	0.267	32..144	
*HbOLE4b*	Scaffold0021_307720	Scaffold0021:307491..307949(+)	0	155	16.68	9.87	106.32	0.220	32..144	
*HbOLE5*	–	Scaffold0103:1654178..1654969(−)	0	156	16.46	10.00	98.72	0.184	30..142	Not annotated
–	–	Scaffold1060:139545..139952(−)	–	–	–	–		–	–	Pseudogene

Genome mining of tung tree, castor bean, and *M. annua* also resulted in five *oleosin* genes each. Among them, the gene model of *RcOLE3*, which was not previously reported [23] and computationally predicted to encode 232 residues (29,794.m003372) [2], was manually optimized on the basis of RNA-seq reads (see Additional file S1). Like *JcOLE2* and *JcOLE5* that are closely located on Chr5, *RcOLE2* and *RcOLE5* are located on the same scaffold, implying a conservative evolution between physic nut and castor been. The hypothesis was further supported by synteny analysis, which revealed one-to-one collinear relationship between physic nut and castor been/tung tree/*M. annua* (Fig. 1).

Although more than one genome assemblies have been available for both cassava and rubber tree, results presented in this study are based on the most complete one: the rubber tree genome of Reyan7–33-97 consists of 7453 scaffolds spanning about 1.37 Gb [8], whereas the cassava genome of AM560–2 consists of 40,044 scaffolds spanning about 582 Mb [6]. Compared with the lack of a high density genetic map in rubber tree, 89.0% of the AM560–2 assembly could be further anchored onto 18 chromosomes on the basis of 22,403 markers available [6]. The search of the cassava genome resulted in eight oleosin-coding loci from seven chromosomes, i.e., Chr1, Chr5, Chr6, and Chr14*–*17 (Table 1 and Fig. 1). For convenience, they were named *MeOLE1a*, *MeOLE1b*, *MeOLE2a*, *MeOLE2b*, *MeOLE3*, *MeOLE4a*, *MeOLE4b*, and *MeOLE5*, respectively. Although only a few ESTs have been available for *MeOLE1a*, the expression of other genes was all supported by RNA-seq reads, which also resulted in optimizing the gene model of *MeOLE1a* where an intron was mis-annotated (Table 1 and Additional file S2). The CDS sequences of three paralogous pairs, i.e. *MeOLE1a*/*b*, *MeOLE2a*/*b*, and *MeOLE4a*/*b*, exhibit a relatively high identity of 65.3*–*78.1% (Table 2). Since these gene pairs are located within syntenic blocks of duplicated chromosomes, they were defined as duplicates derived from the ρ WGD. Synteny analysis further supported one-to-one and one-to-two collinear relationships between physic nut and cassava (Fig. 1), corresponding to the occurrence of the recent ρ WGD and different evolutionary fates of WGD-derived duplicate pairs. Interestingly, close location of *MeOLE2a* and *MeOLE5* on the same chromosome was also observed. Since they are located within syntenic blocks of cassava and physic nut but not that of cassava and poplar, Euphorbiaceae-specific chromosome rearrangement could be speculated after its divergence with Salicaceae.

**Table 2:** *Oleosin* duplicate pairs derived from the ρ WGD in cassava and rubber tree

Duplicate pair	Identity (%)	Ks	Ka/Ks
*MeOLE1a/MeOLE1b*	65.3	0.7135	0.1302
*MeOLE2a/MeOLE2b*	76.7	0.5769	0.2740
*MeOLE4a/MeOLE4b*	78.1	0.4431	0.3616
*HbOLE1a/HbOLE1b*	84.9	0.4095	0.2467
*HbOLE2a/HbOLE2b*	87.6	0.3569	0.1233
*HbOLE4a/HbOLE4b*	88.5	0.2764	0.2053

In rubber tree, by contrast, the *oleosin* family was shown to be relatively complex, which includes eight expressed genes as well as three pseudogenes that are incomplete and without evidence for their expression (Table 1). These eight expressed *HbOLE* genes, which are distributed across seven scaffolds, exhibit one-to-one collinear relationship with that of cassava and thereby were named after their orthologs, i.e., *HbOLE1a*, *HbOLE1b*, *HbOLE2a*, *HbOLE2b*, *HbOLE3*, *HbOLE4a*, *HbOLE4b*, and *HbOLE5* (Fig. 1). Among them, the CDS sequences of three paralogous pairs (i.e. *HbOLE1a*/*b*, *HbOLE2a*/*b*, and *HbOLE4a*/*b*) exhibit 84.9*–*88.5% identity, and the value is relatively bigger than their counterparts in cassava. Correspondingly, the Ks value of three rubber tree duplicate pairs varies from 0.2764 to 0.4095, which is relatively smaller than that in cassava (i.e. 0.4431*–*0.7135) (Table 2), implying a higher rate of gene evolution in the latter. In fact, relatively low Ks values of *OLE* duplicate pairs were also observed in another tree species poplar, varying from 0.1470 to 0.3486 (see Additional file 3). Except for *PtOLE2a*/*PtOLE2b*, duplicate pairs in cassava, rubber tree, and poplar possess a Ka/Ks ratio of less than 1 (from 0.1233 to 0.6223) (Table 2 and Additional file 3), suggesting that their divergence was mainly driven by purifying selection. Notably, *HbOLE2a* and *HbOLE2b* are located on the same scaffold, implying possible species-specific chromosome rearrangement after rubber tree-cassava divergence. However, due to the lack of a high density genetic map, we have no idea whether *HbOLE2a*/*HbOLE2b* and *HbOLE5* are located on the same chromosome as observed in physic nut, castor bean, and cassava.

### Phylogenetic analysis and definition of ortholog groups

**Fig. 2:**
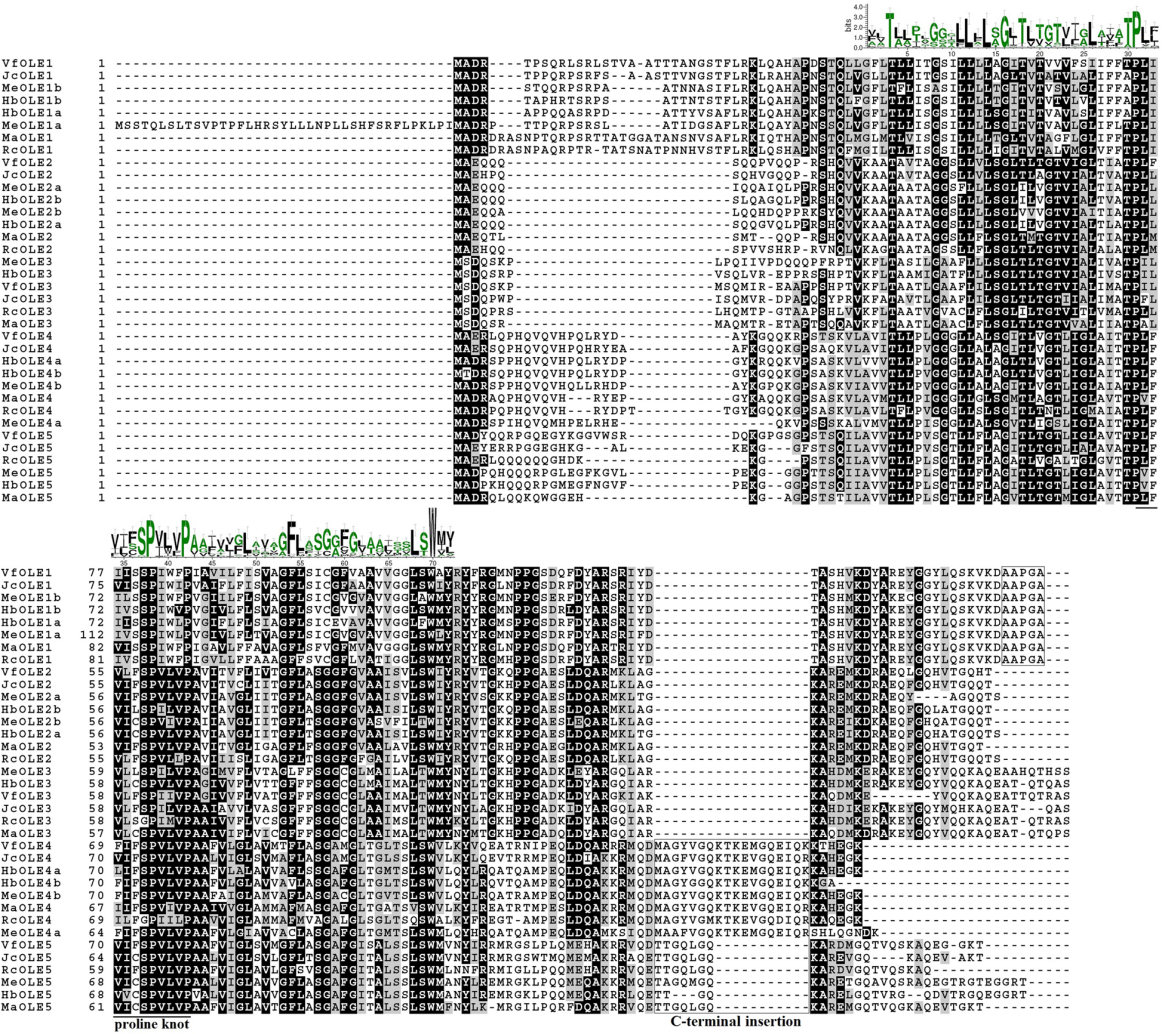
Multiple sequence alignment of oleosin proteins. Identical and similar amino acids are highlighted in black or dark grey, respectively. The SeqLogo of the 72-residue proline knot motif is shown above the alignment, and the PX_5_SPX_3_P pattern is underlined. The C-terminal AAPGA of Clade U and the putative C-terminal insertion of Clade SH are boxed

**Fig. 3:**
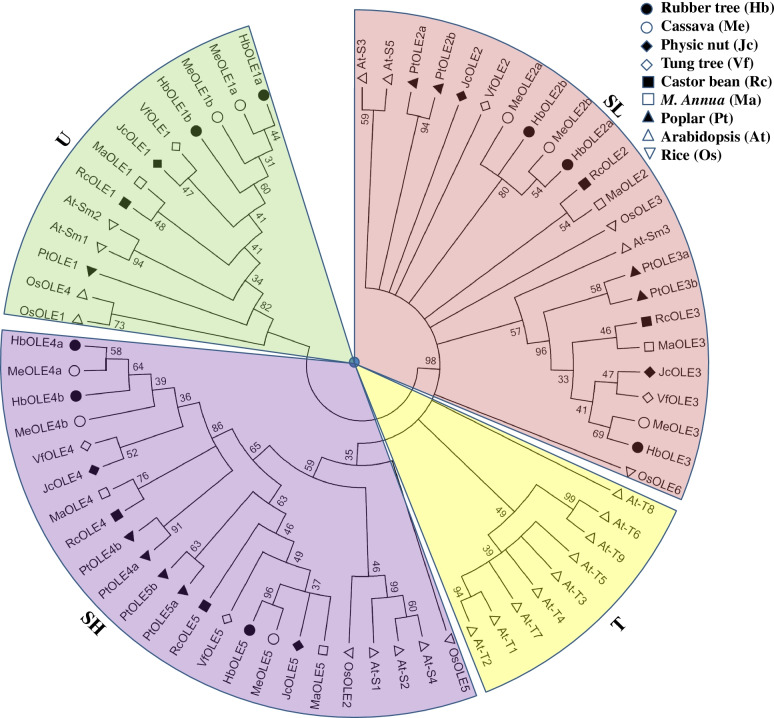
Phylogenetic analysis of oleosins in physic nut, tung tree, castor bean, *M. annua*, cassava, rubber tree, poplar, arabidopsis, and rice. Sequence alignment was performed using MUSCLE and the phylogenetic tree was constructed using bootstrap maximum likelihood tree (1000 replicates) method of MEGA6. Shown are bootstrap values at nodes supported by a posterior probability of ≥30%. The distance scale denotes the number of amino acid substitutions per site. The name of each clade is indicated next to the corresponding group

**Fig. 4:**
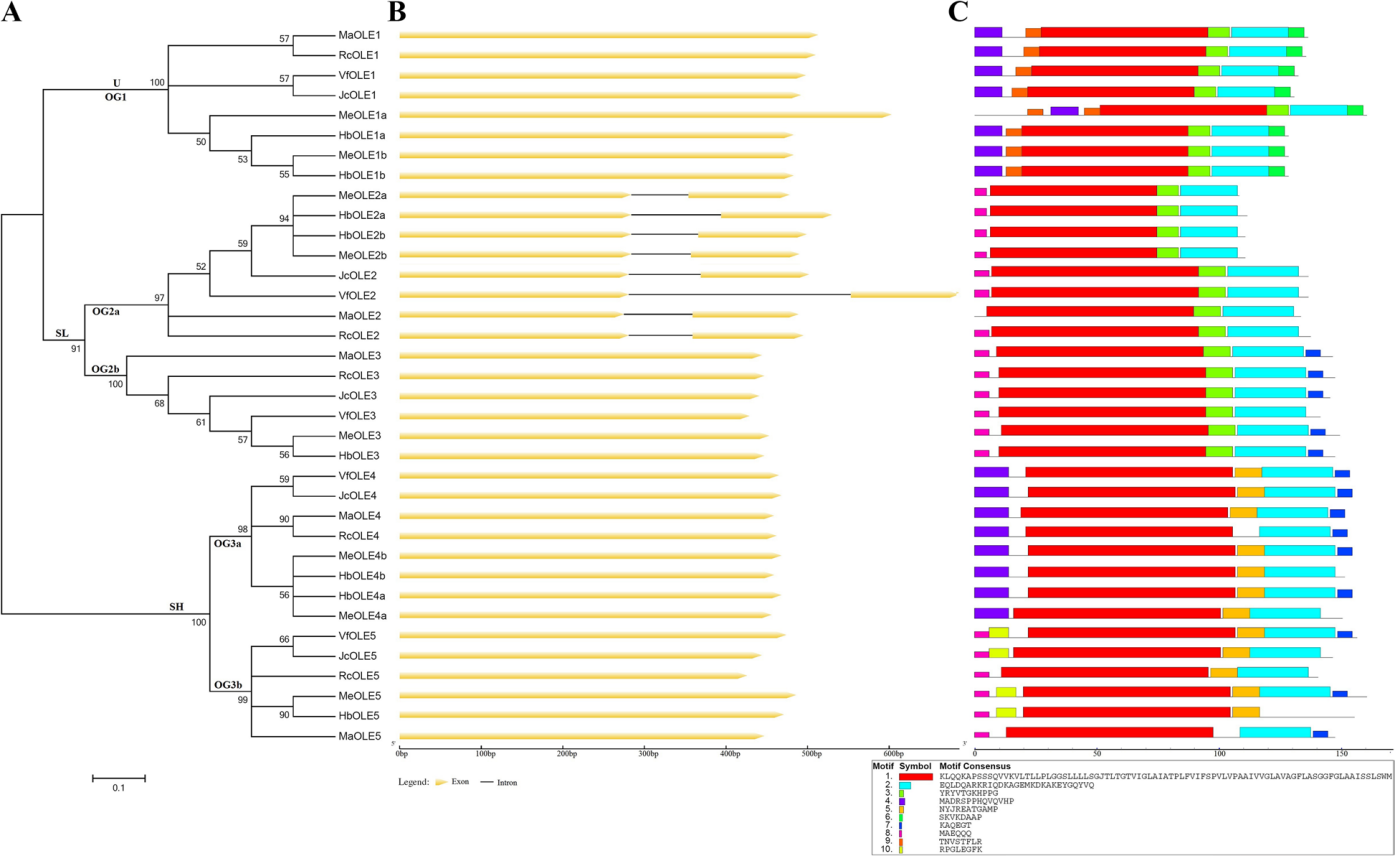
Structural and phylogenetic analysis of *oleosin* genes in physic nut, tung tree, castor bean, *M. annua*, cassava, and rubber tree. **A** Shown is an unrooted phylogenetic tree resulting from full-length oleosins with MEGA6. **B** Shown is the graphic representation of exon-intron structures displayed using GSDS. **C** Shown is the distribution of ten conserved motifs among oleosins, where different motifs are represented by different color blocks as indicated at the bottom of the figure and the same color block in different proteins indicates a certain motif

Although the overall sequence similarity is low (see Additional file 4), 36 oleosins identified in Euphorbiaceae plants all harbor a single oleosin domain (110*–*113 AA), which includes the highly conserved proline knot motif (Fig. 2). To ensure the reliability of phylogenetic analysis, oleosin domain sequences instead of the complete amino acids were used for unrooted tree construction, including five JcOLEs, five RcOLEs, five VfOLEs, five MaOLEs, eight MeOLEs, eight HbOLEs, nine PtOLEs, 17 AtOLEs, and six OsOLEs. As shown in Fig. 3, the tree assigned 68 oleosins into four main clades, i.e. U, SL, SH, and T as described before [19]. Except for T that is arabidopsis-specific, each species was shown to contain at least one member in each other clade. Moreover, both SL and SH have evolved to form two distinct groups in eudicots examined (see more in Fig. 4A). To confirm the result, the BRH method was also employed, which resulted in five ortholog groups, i.e. OG1, OG2a/2b, and OG3a/3b, corresponding to U, SL, and SH, respectively (Table 3). In physic nut, tung tree, castor bean, and *M. annua* that each harbor a single member in OG2a, OG2b, OG3a, and OG3b, OG2a/2b and OG3a/3b exhibit 60.1*–*66.2% and 61.7*–*69.4% sequence similarity, respectively, implying their recent origin. As for other species tested, species or even linage-specific gene expansion and/or loss were found: OG3a is absent from arabidopsis, whereas gene expansion was observed in OG1, OG2a, and OG3b; cassava and rubber tree exhibit same retention patterns, i.e. OG1, OG2a, and OG3a, which are somewhat different from poplar with the expansion of OG2a, OG2b, OG3a, and OG3b (Table 3), a species having experienced one Salicaceae-specific WGD at 60–65 Mya [34].

**Table 3:** Five OGs of the oleosin family based on analyzing nine representative species

Clade	OG	Physic nut	Castor bean	Tung tree	***M. annua***	Cassava	Rubber tree	Poplar	Arabidopsis	Rice
U	OG1	JcOLE1	RcOLE1	VfOLE1	MaOLE1	MeOLE1a	HbOLE1a	PtOLE1	At-Sm1 At-Sm2	OsOLE1 OsOLE4
						**MeOLE1b**	**HbOLE1b**			
SL	OG2a	JcOLE2	RcOLE2	VfOLE2	MaOLE2	MeOLE2a	HbOLE2a	PtOLE2a PtOLE2b	At-S3 At-S5	OsOLE3 OsOLE6
						**MeOLE2b**	**HbOLE2b**			
	OG2b	JcOLE3	RcOLE3	VfOLE3	MaOLE3	MeOLE3	HbOLE3	PtOLE3a PtOLE3b	At-Sm3	
SH	OG3a	JcOLE4	RcOLE4	VfOLE4	MaOLE4	MeOLE4a	HbOLE4a	PtOLE4a PtOLE4b	ND	OsOLE2 OsOLE5
						**MeOLE4b**	**HbOLE4b**			
	OG3b	JcOLE5	RcOLE5	VfOLE5	MaOLE5	MeOLE5	HbOLE5	PtOLE5a PtOLE5b	At-S1 At-S2 At-S4	

Orthologs across different species were identified using the BRH method, and systematic ortholog group names were assigned only when at least one member is found in at least two of species examined, whereas lineage-specific groups present in rubber and cassava are shown in bold

### Exon-intron structures, sequence features, and conserved motifs

As shown in Fig. 4B, the majority of 36 *oleosin* genes identified in this study don’t have introns in the coding region, whereas members in OG2a all harbor a phase 2 intron within the codon of the conserved R just after the proline knot motif and possess a classical GT-AG splice junction. Same exon-intron structure was also observed in poplar (except for a R → G variation in PtOLE2a), by contrast, all *OsOLE* genes are intronless and most *AtOLE* genes contain one to two introns except for intronless genes *At-Sm1/2* and *At-Sm3* in OG1 and OG2b, respectively (see Additional file 5). Compared with a similar length of coding sequences, species-specific insertion and deletion were frequently observed in the intron of OG2a members, which resulted in a variable intron length from 70 bp (*MeOLE2a*) to 272 bp (*VfOLE2*) (Fig. 4B).

Oleosins in examined Euphorbiaceae plants consist of 134–200 AA, and the average of 152 AA is comparable to 150 AA in poplar, 165 AA in rice, and 171 AA in arabidopsis (members in the T clade were excluding for their high variation from 106 to 543 AA, same for other physical and chemical parameters); the theoretical MW varies from 14.00 to 21.72 kDa, and the average of 16.15 kDa is similar to 15.43 kDa in poplar, 16.71 kDa in rice, and 18.43 kDa in arabidopsis (see Table 1 and Additional file 5). Without exception, all these proteins have a *p*I value of greater than 7, varying from 7.97 to 10.96, as well as a high AI value (88.44–119.57) and a GRAVY value of more than 0 (0.131–0.515), indicating their amphipathic property (Table 1). Notably, MeOLE1a has an extended N terminus relative to MeOLE1b and orthologs in other Euphorbiaceae plants, and RcOLE1 harbors a PX_5_GPX_3_P pattern instead of PX_5_SPX_3_P present in most oleosins. Compared with Clades U and SL, a putative fragment insertion was observed in the C-terminal of members in Clade SH, i.e. 18 AA for OG3a and 8 AA for OG3b with the exception of 4 AA for HbOLE5 (Fig. 2). Nevertheless, similar Kyte–Doolittle hydrophobicity plots were observed in all oleosins (Additional file 6).

Conserved motifs were also identified using MEME, which resulted in ten motifs with a range of 6–85 AA. Among them, Motifs 1, 3, and 2 are broadly distributed, which belong to the oleosin domain; Motifs 9 and 6 are OG1-specific, whose functions are unknown and the latter is characterized as a hallmark of the U clade; Motif 7 is widely distributed in OG2b, OG3a, and OG3b, whereas Motif 8 is present in OG2a, OG2b, and OG3b; Motif 5 is present in most members of OG3a and OG3b; Motif 4 is present in OG1 and OG3a, while Motif 10 is only found in OG3b. Species-specific gain or loss of certain motifs was also observed: MeOLE1a has gain one more copy of Motif 9 in its extended N terminus; MaOLE2 has lost Motif 8, whereas VfOLE3, HbOLE4b, MeOLE4a, JcOLE5, RcOLE5, and HbOLE5 have lost Motif 7; RcOLE4 has lost Motif 5, while RcOLE5 and MaOLE5 have lost Motif 10 (Fig. 4C).

### Transcriptional profiling of *oleosin* genes in physic nut, castor bean, rubber tree, and cassava

**Fig. 5:**
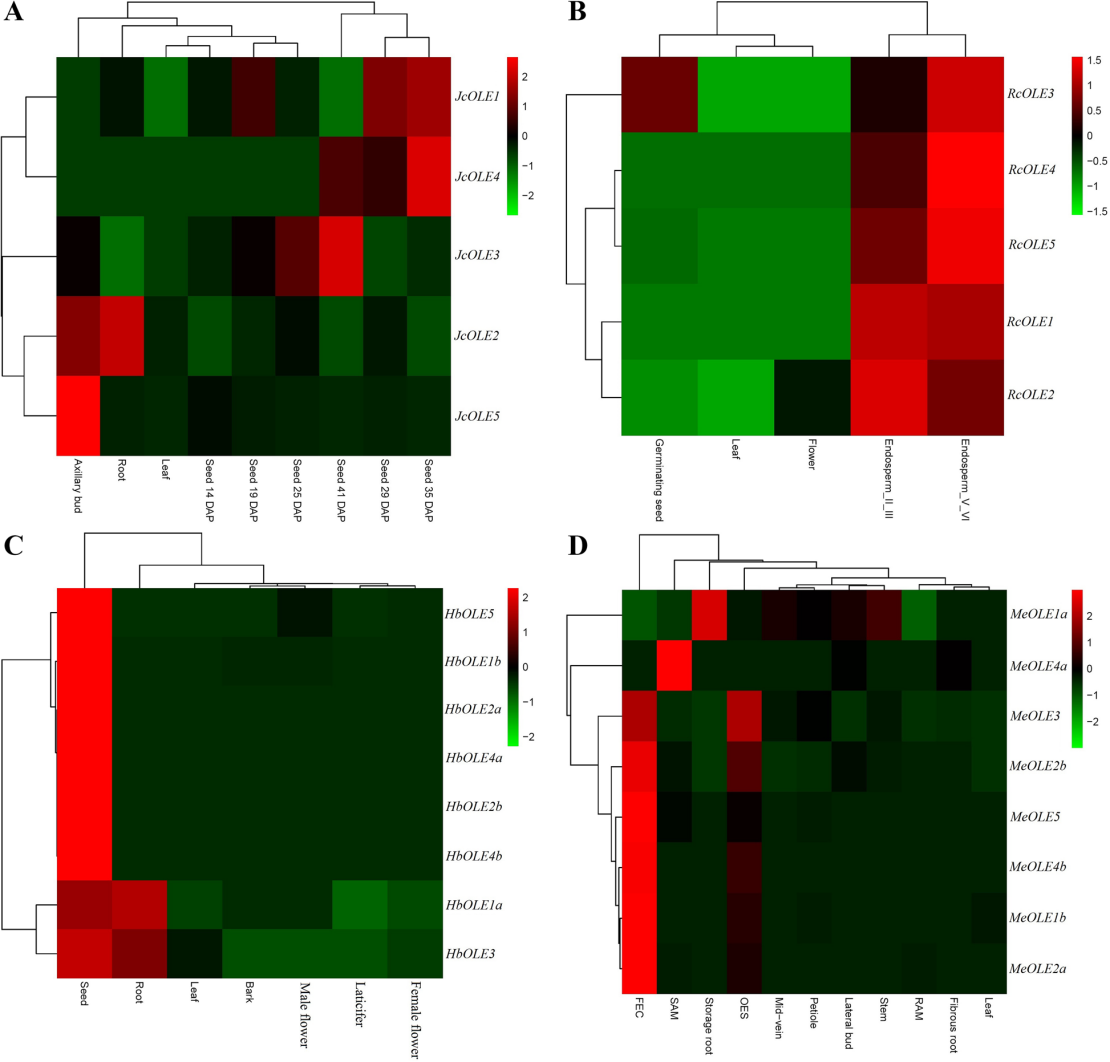
Expression profiles of *oleosin* genes in physic nut, castor bean, rubber tree, and cassava. Color scale represents FPKM normalized log_10_ transformed counts where green indicates low expression and red indicates high expression

To uncover the expression evolution of *oleosin* genes, various tissues and developmental stages were examined in physic nut, castor bean, rubber tree, and cassava, and results are presented in Fig. 5. In physic nut, four tissues (i.e. root, leaf, axillary bud, and seed) and seven stages of developmental seed were investigated. These seven stages, i.e., 14, 19, 25, 29, 35, 41, 45 days after pollination (DAP), were characterized as histodifferentiation, early increase of seed dry-weight, rapid increase of seed coat dry-weight, early increase of kernel dry-weight, rapid increase of kernel dry-weight, late kernel dry-weight increase, and desiccation, respectively. As expected, a considerably high abundance of total *oleosin* transcripts was observed in the latter four stages, coinciding with a rapid increase of oil. Nevertheless, transcripts of *JcOLE1*, *JcOLE2*, and *JcOLE5* were lowly or barely detected, and *JcOLE4* contributes more than 90% of total transcripts. By contrast, total transcripts in three early stages of developmental seed are comparable to that in other tissues, though an apparent tissue-specific expression profile was observed. In leaves, transcripts of *JcOLE1* and *JcOLE5* were barely detected, whereas *JcOLE2* rarely expressed in seeds of 14 DAP. While *JcOLE5* represents the most expressed gene in axillary buds, *JcOLE3* contributes more than 80% of total transcripts in roots, leaves, and seeds of 14, 19, and 25 DAP. In contrast to the ubiquitous expression of *JcOLE3* and *JcOLE4* that peaked in seeds of 41 and 25 DAP, respectively, *JcOLE1*, *JcOLE2*, and *JcOLE5* lowly expressed and exhibit a tissue or developmental stage-specific expression pattern (Fig. 5A).

In castor bean, five tissues or developmental stages were examined, i.e., fully expanded true leaf, male flower, two stages of developmental seed (endosperm I/III and V/VI), and germinating seed. As shown in Fig. 5B, *RcOLE* genes predominantly expressed in endosperm I/III and V/VI, and the total transcripts are 420 and 630 folds more than that in leaves, respectively. By contrast, total transcripts in flowers and germinating seeds were relatively less abundant, which are just six and eight folds more than that in leaves. Whereas the transcript of *RcOLE5* was barely detected in leaves and flowers, other genes were shown to ubiquitously express. *RcOLE4* and *RcOLE1* represent the most and second expressed genes in endosperm I/III and V/VI, respectively, whereas *RcOLE2*, *RcOLE3*, and *RcOLE4* contribute the most transcripts in male flowers, leaves, and germinating seeds, respectively. Unlike *JcOLE1*, *RcOLE1* also expressed in leaves, though the transcript level was relatively low (Fig. 5B).

In rubber tree, seven typical tissues were analyzed, i.e., root, leaf, bark, laticifer, female flower, male flower, and seed, and an apparent seed-predominant expression pattern of *HbOLE* genes was observed. Total transcripts in leaves, bark, and female flowers were shown to be very low, whereas three, five, and 130 folds more were detected in male flowers, roots, and seeds relative to leaves, respectively. All *HbOLE* genes were shown to express in seeds, and *HbOLE2a* contributes the most transcripts. As for other tissues, *HbOLE1a* represents the most expressed gene in roots and bark, whereas *HbOLE3* and *HbOLE5* contribute the most transcripts in leaves and male flowers, respectively (Fig. 5C). Interestingly, no *oleosin* transcripts were detected in the rubber-producing laticifer, though a high number of samples representing primary and secondary laticifers were mined.

In cassava, a total of 11 tissues were investigated, i.e., leaf blade, leaf mid-vein, petiole, stem, shoot apical meristem (SAM), lateral bud, root apical meristem (RAM), fibrous root, storage root, friable embryogenic callus (FEC), and somatic organized embryogenic structure (OES). As observed in above three species, *MeOLE* genes were lowly expressed in leaf, regardless of leaf blade, leaf mid-vein, or petiole. *MeOLE1a* contributes the most transcripts in leaf blade and mid-vein. The expression profile of *MeOLE* genes in petiole is similar to that of mid-vein, where *MeOLE1a* and *MeOLE3* contribute most transcripts. The expression pattern of *MeOLE* genes in stem is similar to that of leaf blade, where *MeOLE1a* and *MeOLE2b* contribute most transcripts. *MeOLE2b* and *MeOLE1a* represent two major isoforms in lateral bud, fibrous root, and SAM, whereas *MeOLE1a* and *MeOLE2b* contribute most transcripts in storage root and RAM. By contrast, total transcripts in FEC and OES were relatively abundant, which are 28 and 13 folds more than that in leaf blade. *MeOLE2b* and *MeOLE4b* contribute most transcripts in FEC, whereas *MeOLE2b* represents the most expressed gene in OES. Overall, *MeOLE1a* and *MeOLE2b* seem to ubiquitously express, while most of other genes are tissue-specific (Fig. 5D).

## Discussion

Increasing evidence supports that widespread WGDs have contributed much to the morphological and physiological diversity in angiosperms [35, 36]. As for two main clades of angiosperms, i.e., monocots and eudicots, it was proven that two WGDs termed τ and γ have played important roles in their diversification, respectively [37, 38]. Moreover, the model monocotyledonous plant rice experienced two additional WGDs named σ and ρ, whereas the model eudicotyledonous plant arabidopsis experienced two successive rounds of WGDs known as β and α, respectively [38, 39]. In the monocot clade, most gramineous plants that provide us food and/or industrial materials possess six oleosin isoforms, the most important structural protein of LDs [19]. By contrast, in the eudicot clade, the gene family was shown to be highly variable, from four members in *Phaseolus vulgaris* to 48 members in rapeseed [19, 25]. In arabidopsis, 17 members representing four clades have been described, i.e., U, SL, SH, and T [19, 22]. The seed-specific SL clade may originally evolve from the universal U clade, and subsequently evolved to form Clades SH and T [19]. The tapetum-specific T clade, which is only found in the Brassicaceae lineage thus far, occupies more than half of total *AtOLE* genes (52.94%). Comparative evolutionary analysis showed that both WGD and single gene duplication have contributed to the expansion of this special gene family, i.e., β WGD (2), α WGD (2), tandem (5), proximal (1), and transposed (1) duplication [35, 40]. Exactly, single gene duplication has driven the expansion of the T clade, whereas β and α WGDs contribute other clades. In rice, WGD (1) as well as transposed (1) and dispersed (1) duplication have contributed to the family expansion [35]. In the Euphorbiaceae lineage, the available genome sequences of several oil-bearing species with or without additional WGDs after the γ event, i.e., castor bean, physic nut, tung tree, *M. annua*, rubber tree, and cassava, provide a good chance to study lineage-specific evolution patterns in this important plant family.

### The ρ WGD contributes to the expansion of the *oleosin* family in cassava and rubber tree

This study presents a first comparative evolutionary analysis of the *oleosin* family in Euphorbiaceae species. In castor bean, *M. annua*, physic nut, and tung tree, four species without recent WGDs, as expected, small numbers of five *oleosin* family genes were respectively identified. By contrast, relatively higher numbers of eight members were found in both cassava and rubber tree, which shared the recent ρ WGD [6]. Phylogenetic analysis divided these oleosins into three clades, i.e. U, SL, and SH, whereas the T clade reported in Brassicaceous plants was not found. Further homology analysis assigned them into five ortholog groups, where both SL and SH clades were shown to include two groups, i.e. OG2a/2b and OG3a/3b. They are more likely to be generated before the radiation of core eudicots, because of (1) relatively high sequence similarity of 60.1*–*69.4% and widely present in core eudicots (e.g. *Carica papaya*, *Theobroma cacao*, *Cucumis sativus*, *Mimulus guttatus*, and *Solanum lycopersicum*), (2) sharing same orthologs in rice as well as *Amborella trichopoda* and *Aquilegia coerulea* (Additional file 7). As we know, *A. trichopoda* represents a sole sister lineage to all other flowering plants [41], and without respective orthologs in this species as well as rice indicates that paralogs may result from duplication events sometime after monocot and eudicot divergence. Without respective orthologs in *A. coerulea*, a member of the early diverging eudicot clade, suggests related duplication events may occur in core eudicots, though the exact time needs further investigation. The typical feature of the U clade or OG1 is the presence of the conserved AAPGA motif at the C-terminal, whereas the SH clade is typical for the presence of putative C-terminal insertion and a relatively higher molecular weight. OG2a differs from OG2b for the presence of the intron immediately after the proline knot motif, whereas OG3b differs from OG3a for a relatively shorter fragment insertion (8 *vs* 18 AA).

Compared with the conservation in four Euphorbiaceae species without recent WGDs, synteny analysis revealed that the *oleosin* family in cassava and rubber tree has expanded along with the ρ WGD in OG1, OG2a, and OG3a. Despite exhibiting same retention patterns, pseudogenes that belong to OG2b and OG3b were only found in rubber tree. In gymnosperms, pseudogenes with apparently nonfunctional oleosin-coding sequences were also identified [19]. This is consistent with a slow genome evolution in long-lived woody perennials and the lack of an efficient elimination mechanism in rubber tree [8, 42]. In poplar and arabidopsis, WGDs also played a predominant role in the expansion of the *oleosin* family, however, evolutionary fates of these duplicated genes seem species-specific. OG2a is the sole group that reserved duplicates in all four examined species, i.e. cassava, rubber tree, poplar, and arabidopsis. In poplar, gene expansion was also found in OG2b, OG3a, and OG3b, while expansion of OG1 and OG3a was found in arabidopsis.

### Structural divergence plays a role in the evolution of *oleosin* family genes in Euphorbiaceae

In addition to gene number variation, sequence and conserved motif analyses reveal structural divergence of members in different ortholog groups or even between paralogs. Compared with OG2b, the ancestor of OG2a may gain one intron sometime after their divergence. Gain or loss of certain motifs between orthologs or even paralogs as shown in Fig. 4 implies their possible functional divergence. A good example is MeOLE1a, which has gain an extended N terminus (39 AA) due to base mutation in the initial 5′ UTR of its encoding gene. The full-length CDS of *MeOLE1a* share 65.3, 68.8, and 65.3% sequence identity with *MeOLE1b*, *HbOLE1a*, and *HbOLE1b*, respectively, however, when the extended sequence was excluded, a considerably higher identity of 81.1, 85.4, and 81.1% was observed. This variation also resulted in higher values of molecular weight (21.72 *vs* 17.28 kDa) and *p*I (10.00 *vs* 9.72) but a relatively lower GRAVY value (0.451 *vs* 0.475). Nevertheless, the AI value and the Kyte–Doolittle hydrophobicity plot are not much changed (Additional file 6). Thereby, further characterization of the actual protein sequence and investigation of its subcellular localization are of particular interest.

### Evolution of *oleosin* family genes was also associated with expression divergence

Expression divergence is also a key mechanism for duplicate pairs to perform same functions in different tissues or developmental stages [43]. Such studies have been reported in model plants. In arabidopsis, a study revealed that 73% of old duplicate pairs and 57% of recent duplicate pairs have diverged in expression [44]. In rice, Yim et al. (2009) found that 57.4% of ∼70 MYA duplicated genes and 50.9% of ∼7.7 MYA duplicated genes have diverged in expression [45]. Comparative analysis of genes encoding aquaporins, respiratory burst oxidase homologs, and Dof transcription factors also revealed expression divergence of paralogous pairs in cassava and rubber tree [13–15, 46]. In this study, similar results were also observed. In cassava, *MeOLE2b* and *MeOLE1a* have evolved to express ubiquitously, whereas transcripts of their paralogs *MeOLE2a* and *MeOLE1b* are usually low and exhibit a tissue-specific expression pattern, though *MeOLE1b* expressed more than *MeOLE1a* in FEC, implying possible neofunctionalisation. Compared with the rare expression of *MeOLE4a* in most tissues tested, *MeOLE4b* preferentially expressed in FEC and OES, implying possible neofunctionalisation or degeneracy. Like *MeOLE1a*, *HbOLE1a* also exhibits a ubiquitous expression pattern with the exception of laticifer, a rubber tree-specific tissue special for rubber biosynthesis and storage [12]. Based on their origin, laticifers could be divided into primary and secondary laticifers, which are derived from procambium and vascular cambium of tree trunk, respectively [47]. No matter what type, laticifers contain a large number of rubber particles that are surrounded by a monolayer of lipids with proteins such as rubber elongation factor (REF) and/or small rubber particle protein (SRPP). Like oleosins, REF and SRPP are two predominant proteins with a small molecular weight of 14.7 and 22.4 kDa in laticifer, respectively [8]. The high abundance of REF/SRPPs and the absence of *oleosin* transcripts support tissue or cell-specific evolution for specialized biological functions. Compared with *HbOLE1a*, in most tissues, the transcript level of *HbOLE1b* is usually lower but five folds more were observed in seed, implying possible subfunctionalisation. Unlike the ubiquitous expression of *MeOLE2b*, both *HbOLE2b* and *HbOLE2a* were shown to express in a few tissues tested, i.e. seed and female flower, and the transcript level of *HbOLE2a* is 27 folds more than that of *HbOLE2b*. Unlike *JcOLE4* and *RcOLE4* that expressed in all tissues examined, the transcripts of *HbOLE4a* and *HbOLE4b* were only detected in seed, where *HbOLE4b* were shown to express considerably more (about 15 folds). Additionally, despite the universal presence of the U clade, the expression level of genes in this group is usually low, however, *RcOLE1* is highly abundant and represents the second most expressed isoform in endosperm I/III and V/VI; in contrast to the constitutive expression of *JcOLE3* that contribute most transcripts in early stages of developmental seed, the transcripts of *JcOLE4* and *RcOLE4* significantly accumulate in latter stages of developmental seed. Thereby, further characterization of their promoters is of particular interest. In fact, *OLE* promoters from maize (*Zea mays*) and oil palm (*Elaeis guineensis*) have successfully been employed to drive key genes to increase oil production [48, 49].

## Conclusions

Taken together, a genome-wide identification and comprehensive comparison of *oleosin* family genes were performed in representative Euphorbiaceae species, resulting in five to eight members representing three clades (i.e. U, SL, and SH) or five ortholog groups. In contrast to the high conservation in castor bean, physic nut, tung tree, *M. annua*, the family expansion observed in cassava and rubber tree was contributed by the recent ρ WGD and gene evolution was associated with both structure and expression divergence. These findings improved our knowledge on lineage-specific evolution of the *oleosin* family in Euphorbiaceae, which provides valuable information for further functional analysis and utilization of key members and their promoters.

## Methods

### Datasets and sequence retrieval

Arabidopsis, poplar, and rice *oleosin* family genes described before (see Additional file 5) were retrieved from TAIR11 (https://www.arabidopsis.org/), Phytozome v12 (https://phytozome.jgi.doe.gov/pz/portal.html), and RGAP7 (http://rice.plantbiology.msu.edu/), respectively. Genomic sequences of tung tree and *M. annua* were downloaded from NGDC (http://bigd.big.ac.cn/gsa) and OSF (https://osf.io/a9wjb/), respectively, whereas genomic sequences of cassava, castor bean, and other representative plants were accessed from Phytozome v12. mRNA sequences such as nucleotides, Sanger expressed sequence tags (ESTs), and RNA sequencing (RNA-seq) reads as well as genomic sequences of rubber tree and physic nut were accessed from NCBI (https://www.ncbi.nlm.nih.gov/).

### Identification and manual curation of *oleosin* family genes

The oleosin domain profile (PF01277) retrieved from Pfam 33.1 (https://pfam.xfam.org/) was used for HMMER (v3.3, http://hmmer.janelia.org/) searches. Gene models of all candidates were manually curated with available mRNAs as described before [12]. To identify pseudogenes and/or gene fragments, the CDS sequences of candidates were further adopted for the BLASTN search [50] of target genome sequences. Presence of the oleosin domain in candidates was checked using MOTIF Search (https://www.genome.jp/tools/motif/), and their gene structures were displayed using GSDS2.0 (http://gsds.cbi.pku.edu.cn/).

### Synteny analysis and definition of ortholog groups

Chromosomal locations of *MeOLE* genes were inferred from the genome annotation [6], while in physic nut, the linkage map with 1208 genetic markers [5] was employed for such purpose by using MAPchart 2.3 [51]. For synteny analysis, duplicate pairs were identified using the all-to-all BLASTp method, and gene colinearity was inferred using MCScanX [52]. Duplication modes such as tandem, proximal, transposed, dispersed, and WGD were defined as previously described [14, 15], and Ks (synonymous substitution rate) and Ka (nonsynonymous substitution rate) of duplicate pairs were calculated using codeml [53]. Orthologs across different species were identified using the BRH method as well as information from synteny analysis, and ortholog groups were defined only when at least one member is found in at least two of species examined.

### Sequence alignment and phylogenetic analysis

Protein multiple sequence alignment was carried out using MUSCLE (http://www.drive5.com/muscle/), and sequence alignment display was performed using Boxshade (https://embnet.vital-it.ch/software/BOX_form.html). Phylogenetic trees were constructed using MEGA 6.0 [54] with the following parameters: the maximum likelihood method, bootstrap of 1000 replicates, and substitution with the Jones-Taylor-Thornton (JTT) model.

### Protein properties and conserved motif analysis

Protein properties were calculated using ProtParam (http://web.expasy.org/protparam/), which include the theoretical molecular weight (MW), isoelectric point (*p*I), aliphatic index (AI), and grand average of hydropathicity (GRAVY). Conserved motifs in oleosins were analyzed using MEME (https://meme-suite.org/meme/tools/meme) with parameters of any number of repetitions, maximum number of 10 motifs, and the width of 6 and 120 residues for each motif.

### Gene expression analysis

Transcript levels of *oleosin* genes were investigated by using transcriptome datasets as shown in Additional file 8, where SRA experiments with seed samples were preferentially selected. As for cassava without seed samples, SRA experiments with most tissue samples were selected. Raw sequence reads in the FASTQ format were obtained using fastq-dump, and quality control was performed using Trimmomatic [55]. Read mapping was carried out using Bowtie 2 [56], and methods of FPKM (Fragments per kilobase of exon per million fragments mapped) and RPKM (Reads per kilobase per million mapped reads) were adopted to determinate relative transcript levels for pair-ended or single-ended samples, respectively [57]. Unless specified, the tools used in this study were performed with default parameters.

## Supplementary Information

**Additional file 1. **The gene model for *RcOLE3*. The coding region is marked with uppercase letters, above which are its deduced amino acids (the oleosin domain is shown in red). The start and stop codons are marked with bold letters.

**Additional file 2. **The gene model for *MeOLE1a*. The coding region is marked with uppercase letters, above which are its deduced amino acids (the oleosin domain is shown in red). The start and stop codons are marked with bold letters.

**Additional file 3. ***Oleosin* duplicate pairs in poplar.

**Additional file 4. **Percent similarity between different *oleosin* family members in physic nut, castor bean, tung tree, *M. annua*, cassava, rubber tree, poplar, arabidopsis, and rice.

**Additional file 5. **Detailed information of *oleosin* family genes present in arabidopsis, poplar, and rice. ^1^ Duplicated modes were determined based on the study of Qiao et al. (2019).

**Additional file 6. **Kyte–Doolittle hydrophobicity plots of oleosins in physic nut, tung tree, castor bean, *M. annua*, cassava, and rubber tree.

**Additional file 7. **Species-specific distribution of five oleosin OGs identified in this study. Orthologs across different species were identified using the BRH method, and systematic ortholog group names were assigned only when at least one member is found in at least two of species examined. Lineage-specific groups present in rubber and cassava are shown in bold. *C. papaya*, *T. cacao*, *C. sativus*, *M. guttatus*, and *S. lycopersicum* are other representatives of core eudicots, whereas *A. coerulea*, rice, and *A. trichopoda* were used as out-groups before divergence of OG2a/2b and OG3a/3b.

**Additional file 8.** Detailed information of transcriptome data used in this study.

## Data Availability

The datasets analyzed during the current study are available in the NCBI SRA repository (https://www.ncbi.nlm.nih.gov/sra/) and detailed accession numbers can be found in Additional file 8.
