# Supplementary material for: Genomic insights into lineage-specific evolution of the oleosin family in Euphorbiaceae
Source: BMC Genomics. 2022 Mar 5;23:178. doi: 10.1186/s12864-022-08412-z (PMC8897914; doi:10.1186/s12864-022-08412-z)
Supplement: Supplementary file 1 — Additional file 1. The gene model for RcOLE3. The coding region is marked with uppercase letters, above which are its deduced amino acids (the oleosin domain is shown in red). The start and stop codons are marked with bold letters. [file 12864_2022_8412_MOESM1_ESM.pdf]

Additional file 1: The gene model for *RcOLE3*. The coding region is marked with uppercase letters, above which are its deduced amino acids (the oleosin domain is shown in red). The start and stop codons are marked with **bold** letters.

1 tttcctcgcgtccacatctcatatacaaacctttcttattttgtacacatttcgagaaga

61 tcactccccactatatcacttcccgtactcacgcccttatgcactcgcctccattgttct

1 M S D

121 ccaagaacaaatcctctccttttctgcttattattaccacacgccgttcaac**ATG**TCTGA

4 Q P R S L H Q M T P G T A A P S H L V V

181 TCAACCAAGATCTCTGCACCAGATGACCCCTGGTACAGCAGCACCCTCTCATCTTGTGGT

24 K F L T A A T V G V A C L F L S G L I L

241 CAAGTTCCTAACCGCAGCCACGGTAGGTGTTGCTTGCTTGTTTCTATCCGGTTTGATCTT

44 T G T V I T L V M A T P L L V L S G P I

301 AACCGGGACAGTGATCACCTTGGTTATGGCCACTCCTCTGTTGGTTCTTTCTGGTCCCAT

64 M V P A A I V V F L V C S G F F F S G G

361 TATGGTCCCTGCTGCAATAGTTGTATTCTTGGTTTGCTCGGGGTTCTTTTTCTCTGGCGG

84 C G L A A I M S L T W M Y K Y L T G K H

421 GTGTGGGTTGGCGGCGATAATGTCTTTAACTTGGATGTACAAGTACTTGACAGGGAAGCA

104 P P G A D K L D Y A R G Q I A R K A H D

481 TCCGCCAGGTGCTGATAAGTTGGACTATGCAAGAGGACAGATAGCTAGAAAGGCTCATGA

124 M K E R A K E Y G Q Y V Q Q K A Q E A T

541 TATGAAGGAGAGAGCTAAAGAATATGGACAGTATGTTCAGCAAAAAGCACAAGAAGCTAC

144 Q T R A S *

601 TCAAACTCGAGCATCT**TAA**tcaactctgatgtagcttttctttgttctcttataactata

661 acttatgtgctgttagttttgttctgttttggtgcatgttcttgatcacattatgtattt

721 gctttttcatttttgtttcttttgtggatgtggttgtttctgatattttgaggatgatt

781 ttcggttaataaaatcttttagtttcgggtaa
