# Supplementary material for: Genomic insights into lineage-specific evolution of the oleosin family in Euphorbiaceae
Source: BMC Genomics. 2022 Mar 5;23:178. doi: 10.1186/s12864-022-08412-z (PMC8897914; doi:10.1186/s12864-022-08412-z)
Supplement: Supplementary file 2 — Additional file 2. The gene model for MeOLE1a. The coding region is marked with uppercase letters, above which are its deduced amino acids (the oleosin domain is shown in red). The start and stop codons are marked with bold letters. [file 12864_2022_8412_MOESM2_ESM.pdf]

Additional file 2: The gene model for *MeOLE1a*. The coding region is marked with uppercase letters, above which are its deduced amino acids (the oleosin domain is shown in red). The start and stop codons are marked with **bold** letters.

1 ctacacgtgtgtgctgatgcatgagttcaacccaactctctttgacatctgtccccaccc

61 cttttcttcatcgatcctatcttcttctcaaccctttgctctctcatttctctcgctttc

1 M A D R P T T P Q R P S R S L

121 ttccgaagcttcctatc**ATG**GCTGACCGTCCTACGACTCCTCAGCGACCTTCAAGATCCT

16 A T I D G S A F L R K L Q A Y A P N S S

181 TGGCTACAATCGATGGCTCTGCCTTTCTACGTAAACTCCAAGCTTATGCTCCCAACTCAT

36 Q L V G F L T L L I S G S I L L L L S G

241 CCCAACTCGTTGGTTTCTTGACTCTCCTCATATCTGGCTCAATTCTTCTCCTCCTTTCAG

56 I T V T V A V L G L I F L T P L I I V S

301 GTATCACTGTCACAGTTGCTGTTCTTGGTCTTATTTTTCTAACTCCTTTGATTATCGTTT

76 S P I W L P V G I V L F L T V A G F L S

361 CAAGCCCAATTTGGCTCCCCGTCGGAATCGTTCTCTTTCTTACGGTTGCTGGGTTTTTGT

96 I C G V G V A V V G G L S W L Y R Y Y R

421 CGATTTGCGGGGTCGGAGTGGCGGTTGTGGGTGGATTGTCGTGGTTGTATAGGTATTATA

116 G M N P P G S D R F D Y A R S R I F D T

481 GAGGGATGAATCCACCCGGTTCCGATCGGTTCGATTATGCTCGAAGCCGGATCTTTGATA

136 A S H V K D Y A R E Y G G Y L Q S K V K

541 CGGCGAGCCATGTGAAGGATTATGCTAGAGAGTATGGTGGATACCTGCAGAGCAAGGTGA

156 D A A P G A *

601 AGGATGCGGCTCCAGGAGCA**TGA**tgatgggtcgggtcgggtcgggtctatagtgggtttg

661 tgtgtgtcttcaaaaatggggttgtttggacgtttgcttattaattaatcaaatttgttg

721 tgtttttatttgtaaataatcatgtatgctttatggatattctttgatgacgtttcgtc

781 gttattggatttgagaatgtgattaattttacccgaa
