# Supplementary material for: Genomic insights into lineage-specific evolution of the oleosin family in Euphorbiaceae
Source: BMC Genomics. 2022 Mar 5;23:178. doi: 10.1186/s12864-022-08412-z (PMC8897914; doi:10.1186/s12864-022-08412-z)
Supplement: Supplementary file 6 — Additional file 6. Kyte–Doolittle hydrophobicity plots of oleosins in physic nut, tung tree, castor bean, M. annua, cassava, and rubber tree. [file 12864_2022_8412_MOESM6_ESM.pdf]

Additional file 6: Kyte–Doolittle hydrophobicity plots of oleosins in physic nut, tung tree, castor bean, *M. annua*, cassava, and rubber tree.

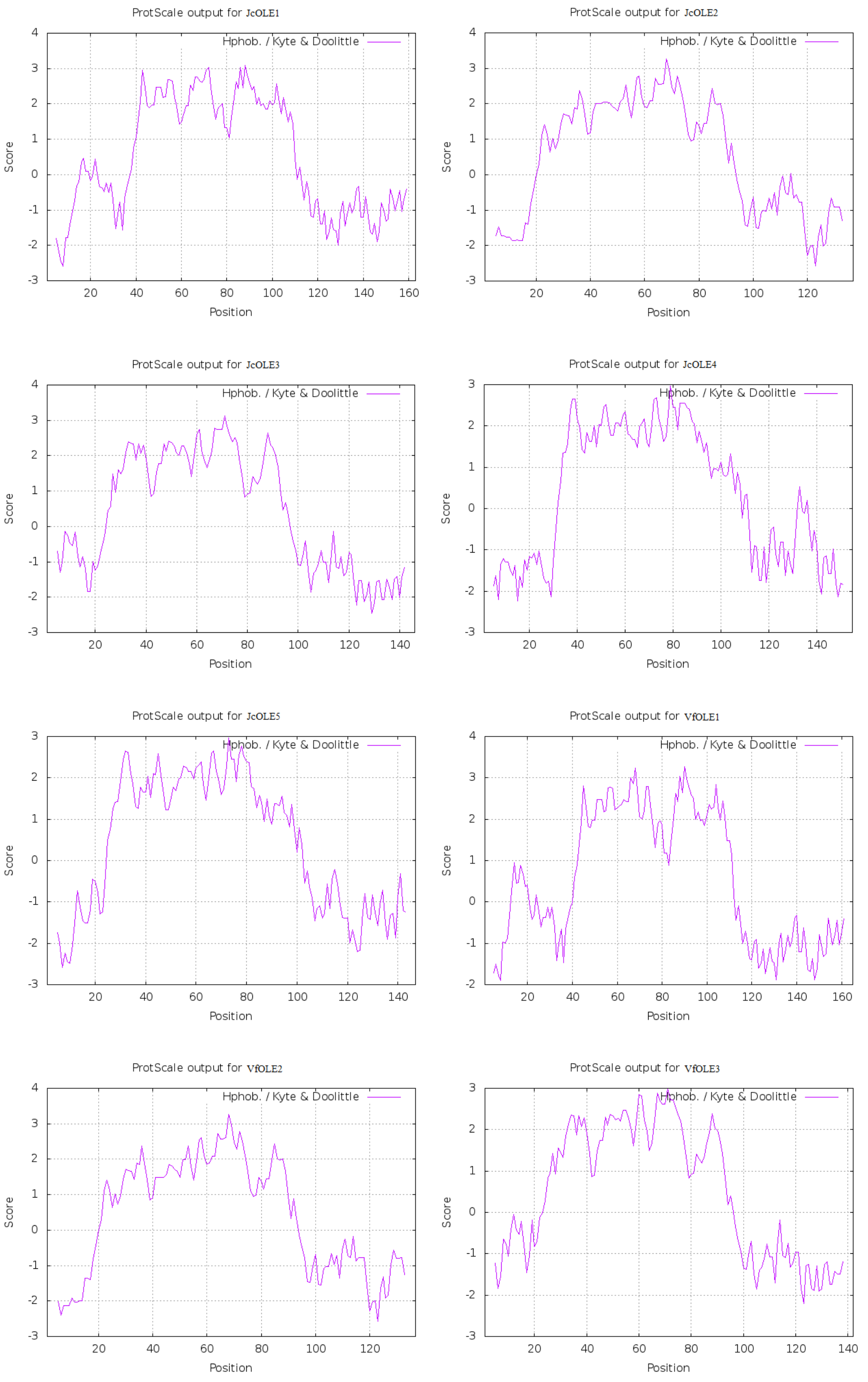

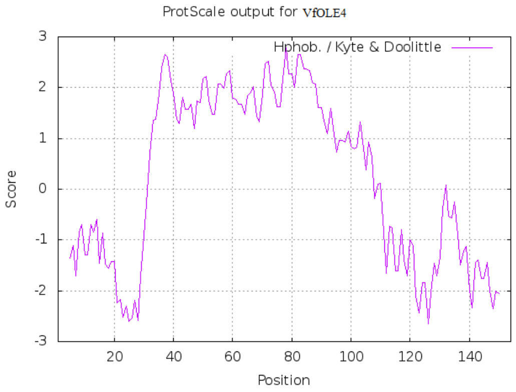
ProtScale output for VfOLE4
Hphob. / Kyte & Doolittle
Score
Position

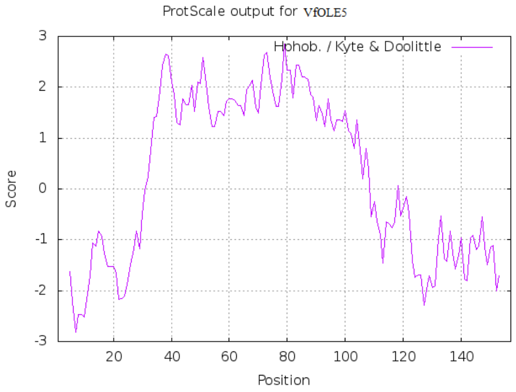
ProtScale output for VfOLE5
Hphob. / Kyte & Doolittle
Score
Position

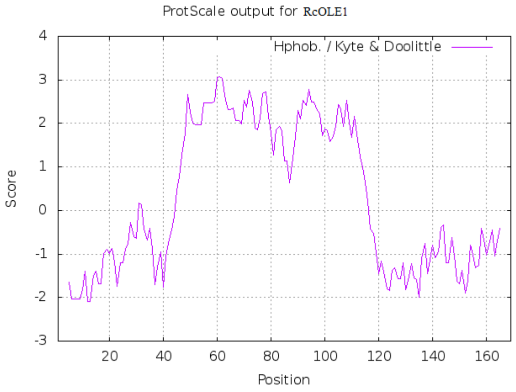
ProtScale output for RcOLE1
Hphob. / Kyte & Doolittle
Score
Position

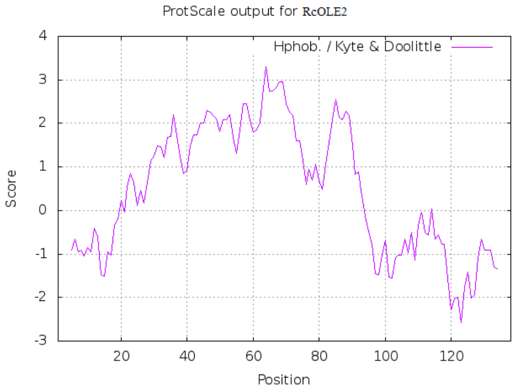
ProtScale output for RcOLE2
Hphob. / Kyte & Doolittle
Score
Position

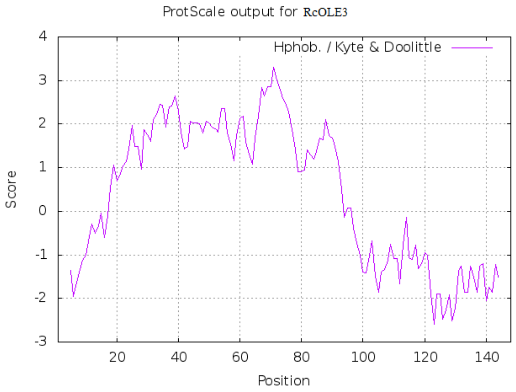
ProtScale output for RcOLE3
Hphob. / Kyte & Doolittle
Score
Position

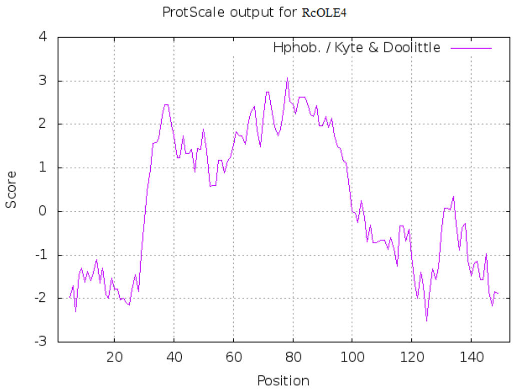
ProtScale output for RcOLE4
Hphob. / Kyte & Doolittle
Score
Position

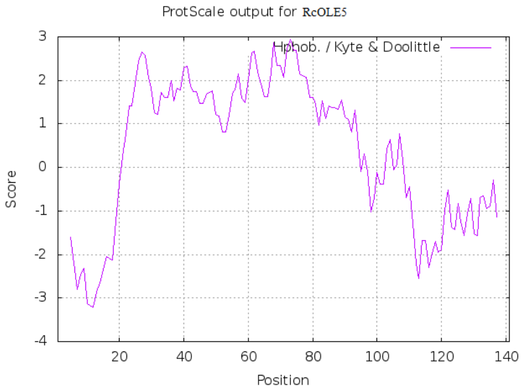
ProtScale output for RcOLE5
Hphob. / Kyte & Doolittle
Score
Position

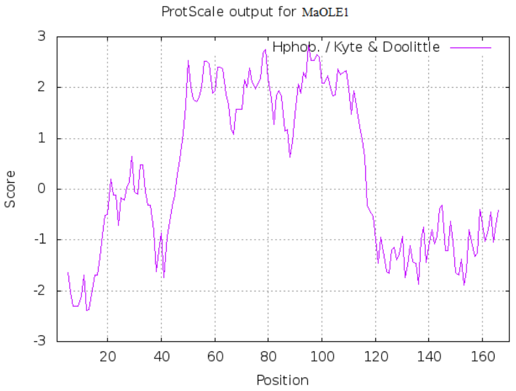
ProtScale output for MaOLE1
Hphob. / Kyte & Doolittle
Score
Position

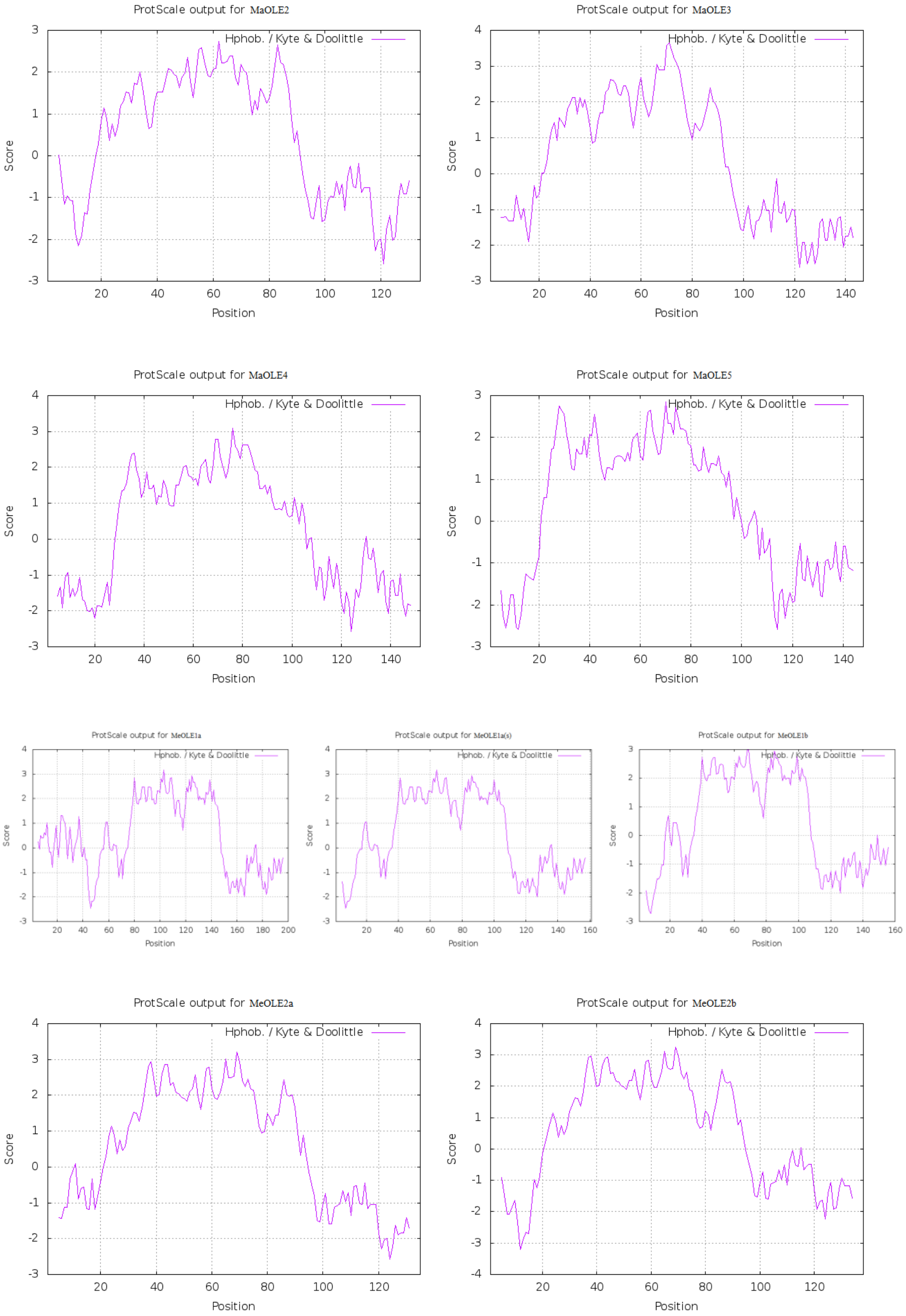

ProtScale output for MaOLE2
ProtScale output for MaOLE3
ProtScale output for MaOLE4
ProtScale output for MaOLE5
ProtScale output for MeOLE1a
ProtScale output for MeOLE1a(s)
ProtScale output for MeOLE1b
ProtScale output for MeOLE2a
ProtScale output for MeOLE2b
Hphob. / Kyte & Doolittle
Score
Position

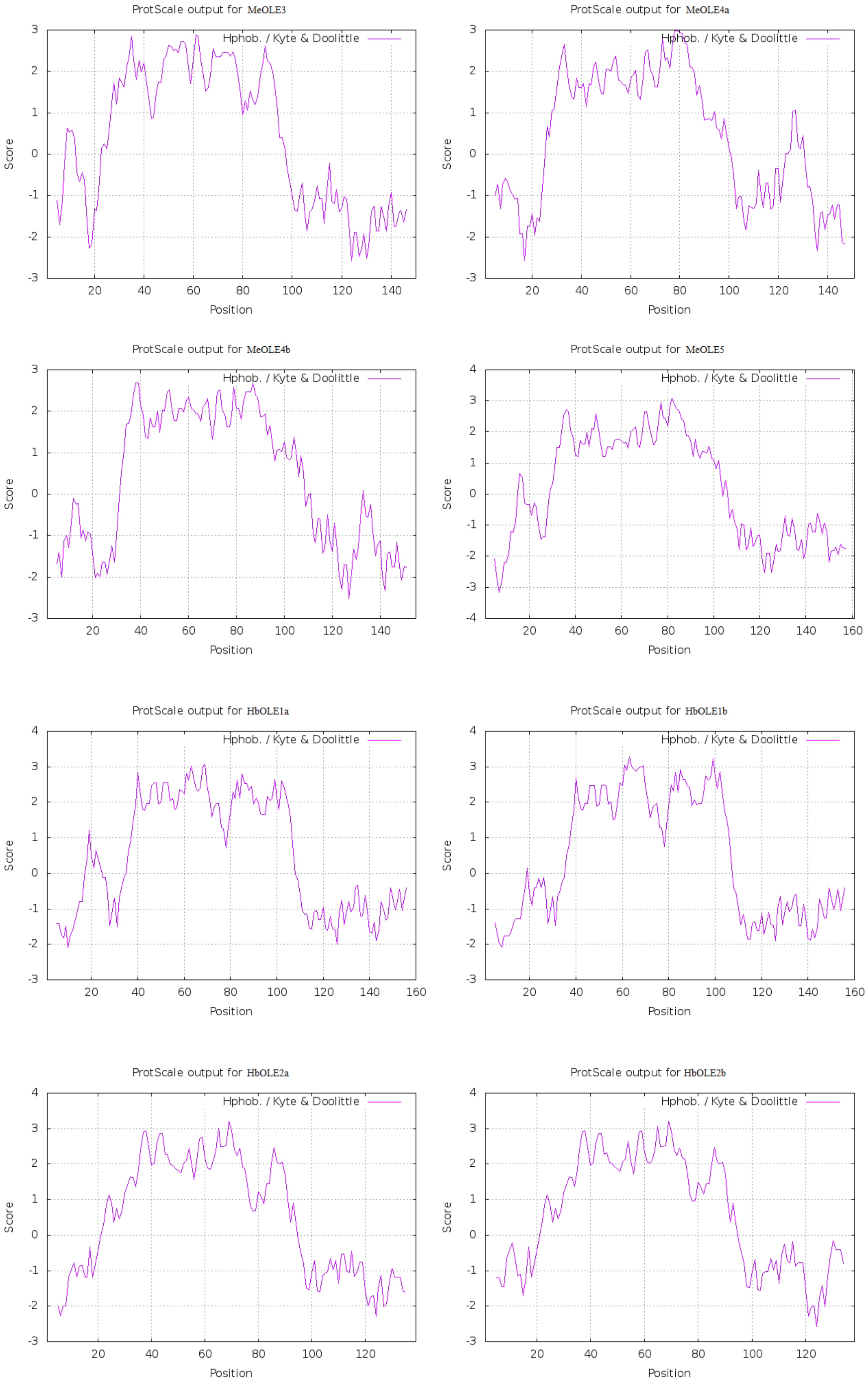
ProtScale output for MeOLE3
Hphob. / Kyte & Doolittle
Score
3
2
1
0
-1
-2
-3
20
40
60
80
100
120
140
Position
ProtScale output for MeOLE4a
Hphob. / Kyte & Doolittle
Score
3
2
1
0
-1
-2
-3
20
40
60
80
100
120
140
Position
ProtScale output for MeOLE4b
Hphob. / Kyte & Doolittle
Score
3
2
1
0
-1
-2
-3
20
40
60
80
100
120
140
Position
ProtScale output for MeOLE5
Hphob. / Kyte & Doolittle
Score
4
3
2
1
0
-1
-2
-3
-4
20
40
60
80
100
120
140
160
Position
ProtScale output for HbOLE1a
Hphob. / Kyte & Doolittle
Score
4
3
2
1
0
-1
-2
-3
20
40
60
80
100
120
140
160
Position
ProtScale output for HbOLE1b
Hphob. / Kyte & Doolittle
Score
4
3
2
1
0
-1
-2
-3
20
40
60
80
100
120
140
160
Position
ProtScale output for HbOLE2a
Hphob. / Kyte & Doolittle
Score
4
3
2
1
0
-1
-2
-3
20
40
60
80
100
120
Position
ProtScale output for HbOLE2b
Hphob. / Kyte & Doolittle
Score
4
3
2
1
0
-1
-2
-3
20
40
60
80
100
120
Position

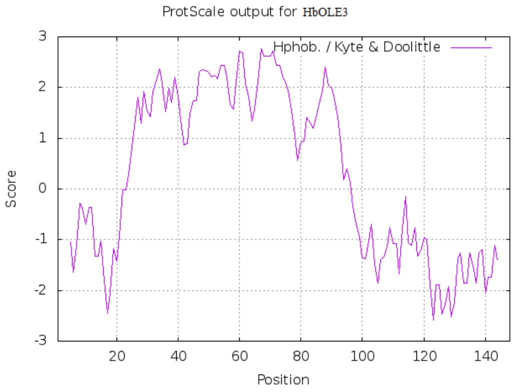
ProtScale output for HbOLE3
Hphob. / Kyte & Doolittle
Score
Position

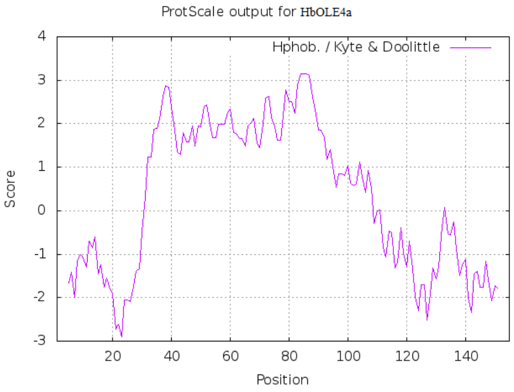
ProtScale output for HbOLE4a
Hphob. / Kyte & Doolittle
Score
Position

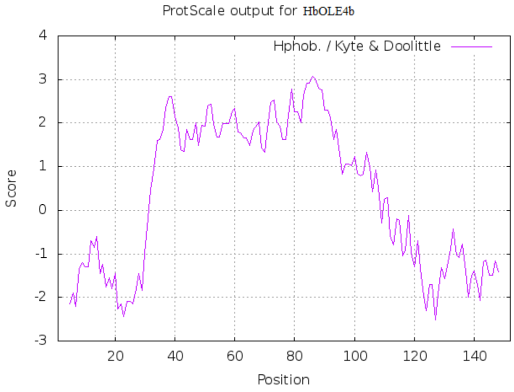
ProtScale output for HbOLE4b
Hphob. / Kyte & Doolittle
Score
Position

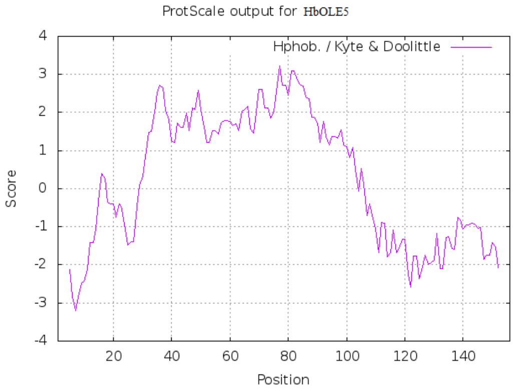
ProtScale output for HbOLE5
Hphob. / Kyte & Doolittle
Score
Position
